# Impact of Severe Obesity and Weight Loss on Systolic Left Ventricular Function and Morphology: Assessment by 2-Dimensional Speckle-Tracking Echocardiography

**DOI:** 10.1155/2016/2732613

**Published:** 2016-02-23

**Authors:** Sevda Karimian, Jürgen Stein, Boris Bauer, Claudius Teupe

**Affiliations:** ^1^Department of Internal Medicine-Cardiology, Hospital Sachsenhausen, 60594 Frankfurt, Germany; ^2^Department of Internal Medicine-Gastroenterology, Hospital Sachsenhausen, 60594 Frankfurt, Germany; ^3^Department of Radiology, Hospital Sachsenhausen, 60594 Frankfurt, Germany

## Abstract

Obesity is associated with an increased risk of heart failure. Little is known about the impact of dietary changes on the cardiac sequelae in obese patients. Twenty-one obese subjects underwent a 12-week low calorie fasting phase of a formula diet. Transthoracic two-dimensional speckle-tracking echocardiography was performed to obtain systolic left ventricular strain before and after weight loss. Body mass index decreased significantly from 38.6 ± 6.2 to 31.5 ± 5.3 kg/m^2^, and the total percentage fat loss was 19%. Weight reduction was associated with a reduction in blood pressure and heart rate. Left ventricular longitudinal global peak systolic strain was in the lower normal range (−18.7 ± 3.2%) before weight loss and was unchanged (−18.8 ± 2.4%) after 12 weeks on diet with substantial weight loss. Also, no significant change in global radial strain after weight loss was noted (41.1 ± 22.0 versus 43.9 ± 23.3, *p* = 0.09). Left atrial and ventricular dimensions were in normal range before fasting and remained unchanged after weight loss. In our study obesity was associated with normal systolic left ventricular function. A 12-week low calorie diet with successful weight loss can reduce blood pressure and heart rate. Systolic left ventricular function and morphology were not affected by rapid weight reduction.

## 1. Introduction

Obesity is a major public health problem, with a prevalence of 10% to 20% in Western Europe [1]. Obesity is characterized by both a large increase in body weight and an increase in body fat exceeding standard measures [2]. Currently, obesity is classified on the basis of body mass index (BMI), and patients with BMI > 30 kg/m^2^ are considered obese. A BMI > 30 kg/m^2^ has a negative impact on health and is associated with premature atherosclerosis, increased risk of myocardial infarction, and heart failure. Severe obesity is associated with decreased survival, largely because of cardiovascular deaths [3, 4].

Most echocardiographic studies assessing left ventricular (LV) systolic function in obese patients used ejection phase indices like LV ejection fraction and LV fractional shortening. They reported normal or hyperdynamic LV systolic function and no or little differences in lean and obese subjects [5, 6]. Impaired LV systolic function in persons with obesity was only found in the presence of coexistent heart disease. Adverse loading conditions and duration of obesity may also contribute to LV systolic dysfunction [7]. More recent echocardiography technics like tissue-Doppler and strain imaging have revealed evidence of subclinical LV systolic dysfunction in subjects with obesity, even in the presence of normal LV ejection phase indices [8, 9].

Alterations in cardiac function have also been shown to be reversible with weight reduction strategies [10, 11]. Weight loss after interventions like bariatric surgery and dietary weight reduction can be beneficial to cardiovascular structure and function and result in a decrease in cardiovascular risk and total mortality [12–14].

The purpose of this study was to examine systolic left ventricular function and the effect of diet-induced weight loss in patients with severe obesity by transthoracic two-speckle-tracking echocardiography (STE) with LV strain imaging.

## 2. Methods

The study was approved by the ethics committee of the State Medical Council of Hessen, Germany (approval number FF 112/2015). Informed consent was obtained from each patient prior to the study participation.

### 2.1. Patient Population and Study Design

**Figure 1:**
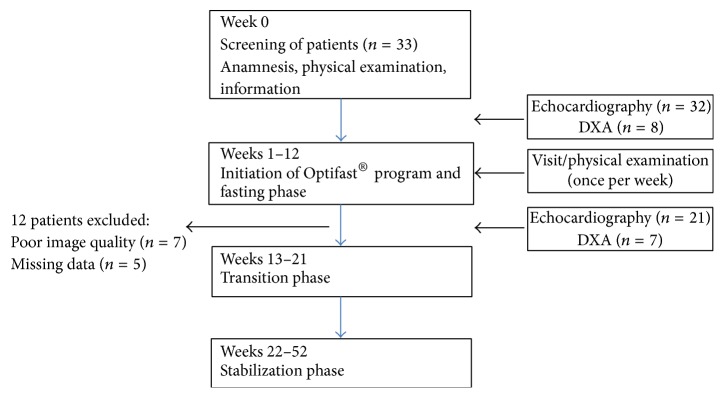
Study flow diagram. DXA: dual energy X-ray absorptiometry.

Initially, 33 patients were included in the study. All patients were participants in the OPTIFAST®-52 diet program (Nestlé Health Science, Frankfurt, Germany), which ran for a total of 52 consecutive weeks. The specifics of the program included a fasting phase of 12 weeks with ingestion of food restricted to formula products with a high protein content. The total calorie intake in this phase is approximately 800 kcal per day. A subsequent transitional phase and a final stabilization phase aim to introduce the patients to a balanced diet. For the duration of the program, all patients received psychological, physiotherapeutic, medical, and nutritional care. Finally his study enrolled 21 patients. Twelve patients were excluded from the analysis (Figure 1).

Inclusion criteria were as follows: age ≥18 and ≤65 years, BMI > 30 kg/m^2^, secondary and accompanying conditions requiring treatment such as hypertension, hypercholesterolemia, diabetes mellitus, and illnesses of the locomotor system. The necessity for participation in the program was documented by a medical certificate. All patients had made repeated (at least three) unsuccessful attempts to reduce weight. Exclusion criteria were cardiac arrhythmia, recent myocardial infarction, severe (malign) general illnesses, pregnancy and nursing, or a documented eating disorder.

Clinical measurements were performed once per week. Before and after the fasting phase, weight and height were recorded in order to calculate the respective BMI. Echocardiography was performed in week 1 and in week 12. Total body composition and fat content was also measured using the dual energy X-ray absorptiometry (DXA) in week 1 and in week 12.

### 2.2. Echocardiography

Echocardiography was performed using a commercially available ultrasound machine (Vivid 7, GE Healthcare, Horten, Norway) and a 3.5 MHz (3S-RS) transducer. Patients were examined in a resting, left-lateral position and, for specific parts of the examination, in end-expiration by an experienced examiner. All images were obtained from standard parasternal and apical position using 2D and M-mode echocardiographic techniques. LV ejection phase indices like LV ejection fraction and fractional shortening were calculated from parasternal M-mode measurements.

Two-dimensional STE analysis was performed offline using dedicated automated software (EchoPAC PC, version 6.0, GE Healthcare, Horten, Norway). From the apical and parasternal short-axis datasets, one cardiac cycle was selected for subsequent analysis. 2D strain was measured as previously described [15, 16]. The endocardial border at end-systolic frame was manually traced. A region of interest was then drawn to include the entire myocardium. The software divided the LV into six segments and performed speckle-tracking analysis throughout one cardiac cycle. Finally, the software automatically created the time-domain LV strain curves in six segments, from which end-systolic strain was calculated. Global longitudinal peak systolic strain (GLPS) was defined as the averaged longitudinal strains at end-systole in 18 segments from three apical views. Global radial strain (GRS) was defined as averaged radial strain at end-systole in 18 segments from three levels of short-axis views.

### 2.3. Dual Energy X-Ray Absorptiometry (DXA)

DXA is a common measurement of human body composition based on a three-compartment model. It divides the body into total body mineral, fat-free soft mass, and fat tissue mass. Body composition was evaluated by DXA with the use of a Hologic densitometer (QDR Discovery Wi, Hologic Inc., Bedford, USA). Measurements were taken with the patient lying in the supine position on the scanning table. Scanning time was approximately 20 minutes. By means of the appropriate software, information on the body composition was obtained from the whole body scan.

### 2.4. Statistical Analysis

The results are expressed as mean ± standard deviation (SD) for continuous variables. For the descriptive statistics, the arithmetic mean, SD, median, minimum and maximum, and first and third quartiles were calculated. For the comparison of two measurements at different times, the Wilcoxon Matched Pairs Test was applied for paired samples. Statistical analyses were performed using a statistical software package (BiAS for Windows, Version 11.0). *p* values < 0.05 were considered statistically significant.

## 3. Results

### 3.1. Clinical Characteristics

**Figure 2:**
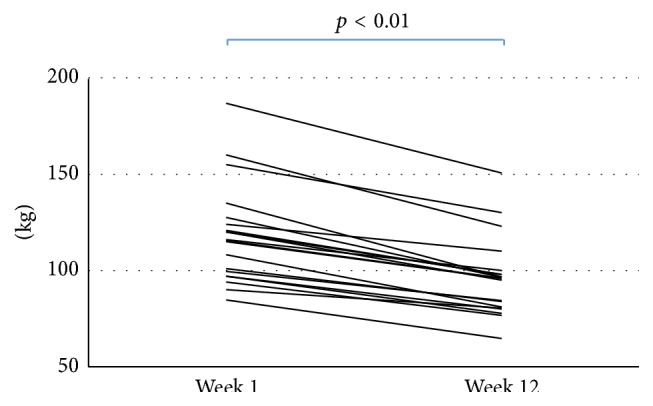
Individual weight loss after 12 weeks on diet.

The clinical characteristics of the patients are shown in Table 1. Body weight decreased significantly from week 1 to week 12 of the weight loss program (Figure 2). The mean percentage weight loss was 19 ± 5% of the baseline weight. BMI was also significantly reduced from 38.6 ± 6.2 to 31.5 ± 5.3 kg/m^2^. Sex and age did not have a significant impact on weight reduction.

The number of patients with arterial hypertension defined as blood pressure >140 mmHg systolic and >90 mmHg diastolic decreased from 9 patients to 1 patient. Mean systolic blood pressure was significantly reduced from 148 ± 16 to 130 ± 17 mmHg and diastolic blood pressure from 95 ± 14 to 82 ± 9 mmHg. The mean heart rate also decreased significantly from 94 ± 16 to 82 ± 9 beats per minute, respectively.

### 3.2. DXA Findings

Body composition was evaluated by DXA in 8 patients. Total body fat mass decreased by 19% (mean) from 50.9 ± 20.9 kg to 41.1 ± 16.9 kg. Changes in fat distribution resulting from the 12 week fasting phase are reported in Table 2.

### 3.3. Echocardiographic Findings

Complete echocardiographic data sets were acquired in 21 patients. Image quality was compromised by high body weight. Grading of the image quality before weight loss was good in 9, moderate in 7, and poor in 5 patients. After weight loss, image quality improved to good in 11, moderate in 7, and poor in 3 patients.

LV ejection phase indices, LV ejection fraction and fractional shortening, were within normal range at baseline and remained unchanged after weight loss (Table 3).

**Figure 3:**
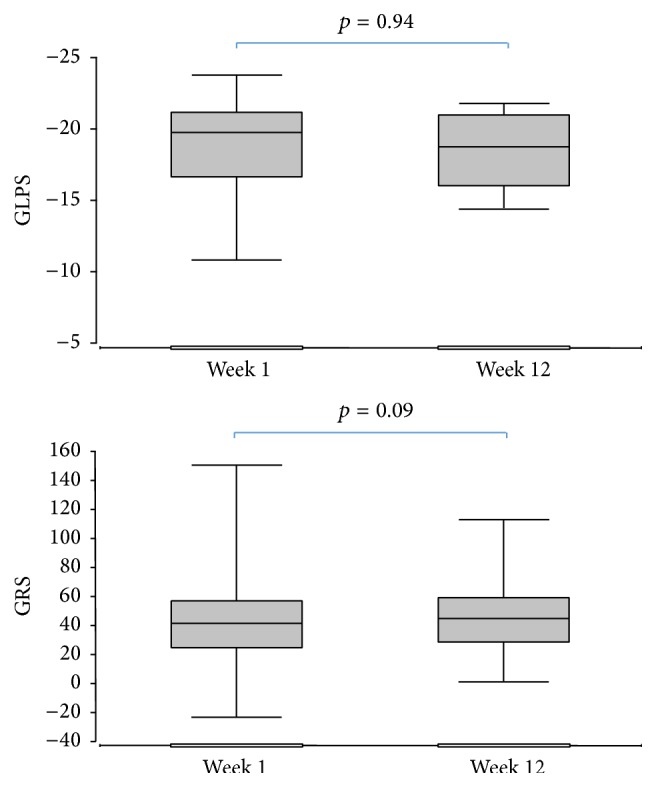
Global longitudinal peak systolic strain (GLPS) and global radial strain (GRS) before and after weight loss.

GLPS at baseline was −18.7 ± 3.2 in week 1 and −18.8 ± 2.4 in week 12 (Figure 3). The difference was not significant. No significant correlation was noted between GLPS and BMI (*r* = −0.36, *p* = ns). We found a weak but not significant correlation between percentage weight loss and increase in GLPS (*r* = 0.39, *p* = ns). Also, no significant change in GRS after weight loss was noted (41.1 ± 22.0 versus 43.9 ± 23.3, *p* = 0.09).

The left atrial diameter (anterior-posterior) measured in the parasternal long-axis view was mildly enlarged at baseline. Weight reduction after 12 weeks of diet was associated with a decrease in left atrial size, but the difference was not significant (Table 3). Left atrial volume, LV wall thickness, and LV end-diastolic diameter were within normal range at baseline and remained unchanged after weight loss.

## 4. Discussion

Obesity is associated with structural and functional abnormalities of the heart. Previous studies have reported the presence of left ventricular diastolic dysfunction. Variable findings have been reported with respect to systolic function in obese subjects, including preserved or mildly reduced left ventricular function. Interestingly, these alterations have been shown to be reversible with weight reduction therapies [10, 17]. Echocardiography is an important tool to provide an estimate of LV function. LV ejection fraction appears to be clinically the most relevant parameter. However, LV ejection fraction has some limitations. The most important, it is not sensitive enough to detect subtle alterations in the contractile function and therefore not suitable for detecting subclinical myocardial damage [18]. In some studies, tissue-Doppler echocardiography has been able to show the presence of subclinical systolic contractile abnormalities, although the left ventricular ejection fraction remained within the normal range [8, 9, 19–22]. Two-dimensional STE is a new gray-scale based technique that provides angle-independent measurements of myocardial deformation and has the potential for quantitative assessment of systolic left ventricular function [18, 23]. Speckles are produced as a result of the scatter of the ultrasound beam by the tissue. By tracking frame by frame the displacement of the speckles during the cardiac cycle, strain can be rapidly measured offline after adequate image acquisition. From this data, software automatically resolves the magnitude of myocardial deformation in different directions and generates strain. Strain is the percentage change in the length of a myocardial segment during a cardiac cycle and has the unit %.

In the present study, LV ejection phase indices, LV ejection fraction and fractional shortening, were normal at baseline and unchanged at follow-up. Also, systolic left ventricular function quantified by GLPS and GRS was normal. We found GLPS and GRS values in a range comparable to values defined as normal in a large meta-analysis including 2,597 subjects from 24 studies [24]. Twelve weeks of the diet had no significant impact on GLPS and GRS. Our findings indicated preserved systolic function before and after weight loss. Other studies in obese individuals without cardiac disease reported similar LV ejection fraction but worse systolic mechanics as assessed by GLPS when compared with nonobese controls [25–27]. A recent study by Leung et al. found a significant improvement in both systolic and diastolic myocardial function measured by longitudinal strain in obese patients with type 2 diabetes and weight loss 9 months after sleeve gastrectomy [28]. A behavioural intervention program including dietary restrictions and exercise training in obese patients found an increase in strain measured by tissue-Doppler myocardial imaging in patients with significant reduction of weight after 6 months [29]. In contrast to other studies we could not find a significant increase in GLPS or GRS after weight loss. This might be due to a shorter follow-up period, a lower rate of concomitant diseases, and different weight loss strategies [30, 31].

In moderate-to-severe cases of obesity, increased cardiac output as a result of the high metabolic activity of excessive fat may lead to left atrial and ventricular dilation, increased LV wall stress, and compensatory (eccentric) LV hypertrophy [32, 33]. In the present study, mean LV diameter and thickness of the LV wall were normal at baseline and remained unchanged after 12 weeks on the diet. An echocardiographic study in obese patients with a BMI of 50.0 ± 1.4 who underwent bariatric surgery reported a decrease in LV dilatation and hypertrophy after a longer follow-up period of 13 ± 4 months [34]. Other studies have also shown a decrease in LV mass and size one to three years after weight reduction interventions [7, 35, 36]. We found no change in left atrial volume and diameter after weight loss. Similar findings were reported in patients with obesity after gastric bypass surgery [36, 37]. Lifestyle and diet therapies have demonstrated some cardiovascular benefits [38]. The data from a number of recent meta-analyses, however, suggests that bariatric operations result in larger benefits to cardiovascular risk parameters than other weight loss therapies [38]. However, no randomized controlled trials currently exist comparing the effects of bariatric surgery with standardized nonsurgical treatments, specifically focusing on cardiovascular end points.

The present study did have some limitations that deserve comment. The small number of the study groups limits interpretation of the results, in particular *p* values. We were unable to demonstrate cardiac changes due to obesity such as LV hypertrophy and enlargement or atrial dilatation as described by other authors. However, patients in our group were relatively young (mean age 43 years) in terms of the risk of other causes of structural cardiac abnormalities. GLPS measured by STE in order to determine systolic function is in part heart rate and blood pressure-dependent and is subject to change within any given patient. Finally, when studying people with obesity, echocardiographic measurements may be error-prone or imprecise.

## 5. Conclusion

In our study, obesity was not associated with systolic dysfunction measured by STE with strain imaging. A 12-week low calorie diet had no effect on systolic function. Dimensions of left atrial and ventricular structures were normal at baseline and not altered after weight loss. Diet with successful weight reduction goes along with reduction in blood pressure and heart rate.

## Figures and Tables

**Table 1:** Clinical characteristics (*n* = 21).

Sex (female/male)	14/7
Age (years)	43.1 ± 7.1
Weight (kg)	118.4 ± 25.5
Height (cm)	174.3 ± 10.8
Body mass index (kg/m^2^)	38.6 ± 6.2
Systolic BP (mmHg)	148 ± 16
Diastolic BP (mmHg)	95 ± 14
HR (beats per minute)	94 ± 16
Hypertension	9 (43%)
Hyperlipidaemia	3 (14%)
Diabetes mellitus	3 (14%)
Smoking	3 (14%)

Data are presented as mean ± SD or numbers (%).

BP: blood pressure; HR: heart rate.

**Table 2:** Changes in fat mass before and after weight loss (*n* = 8).

	Fat mass (kilograms)		Fat loss (%)
	Week 1	Week 12	
Left arm	2.4 ± 0.7 (50%)	1.8 ± 0.7 (50%)	25%
Right arm	2.3 ± 0.8 (48%)	1.8 ± 0.7 (46%)	22%
Trunk	30.1 ± 15.9 (46%)	23.8 ± 11.7 (42%)	21%
Left leg	7.5 ± 2.9 (43%)	6.2 ± 2.4 (42%)	17%
Right leg	7.3 ± 3.0 (43%)	6.3 ± 2.6 (42%)	14%
Head	1.3 ± 0.2 (25%)	1.2 ± 0.2 (26%)	8%

Total	50.9 ± 20.9 (44%)	41.1 ± 16.9 (42%)	19%

Data is presented as mean ± standard deviation or percentage of fat (%).

**Table 3:** Cardiac dimensions and LV ejection phase indices before and after weight loss.

	Week 1	Week 12	*p* value
LA diameter (mm)	41 ± 5	39 ± 9	0.62
LA volume (mL)	53 ± 17	59 ± 20	0.37
LV end diastolic diameter (mm)	50 ± 4	50 ± 4	0.46
LV posterior wall (mm)	11 ± 2	11 ± 1	0.79
Interventricular septum (mm)	11 ± 1	11 ± 1	0.79
AO diameter (mm)	31 ± 4	30 ± 4	0.8
LV ejection fraction (%)	68 ± 10	71 ± 8	0.45
LV fractional shortening (%)	38 ± 9	41 ± 7	0.45

LA: left atrium, LV: left ventricle, and AO: aorta.

Data is presented as mean ± standard deviation.
